# Assembly of nuclear dimers of PI3K regulatory subunits is regulated by the Cdc42-activated tyrosine kinase ACK

**DOI:** 10.1016/j.jbc.2022.101916

**Published:** 2022-04-13

**Authors:** Natasha S. Clayton, Millie Fox, Jose J. Vicenté-Garcia, Courtney M. Schroeder, Trevor D. Littlewood, Jonathon I. Wilde, Kadalmani Krishnan, Murray J.B. Brown, Claire Crafter, Helen R. Mott, Darerca Owen

**Affiliations:** 1 Department of Biochemistry, University of Cambridge, Cambridge, United Kingdom; 2 GlaxoSmithKline Medicines Research Centre, Screening and Compound Profiling, Stevenage, Herts, United Kingdom; 3 Bioscience, Research and Early Development, Oncology R&D, AstraZeneca, Cambridge, United Kingdom

**Keywords:** activated Cdc42 kinase, cancer, Cdc42, nuclear signaling, p110-independent p85, p85 dimers, PI3Kinase, protein degradation, protein phosphorylation, tyrosine kinase, ACK, activated Cdc42-associated kinase, AR, androgen receptor, caACK, constitutively active ACK, cSH2, C-terminal SH2 domain, DMEM, Dulbecco's modified Eagle's medium, EGF, epidermal growth factor, GAP, GTPase-activating protein, nSH2, N-terminal SH2 domain, PI3K, phosphoinositide 3-kinase, SH3, Src homology-3, SH2, Src homology-2

## Abstract

Activated Cdc42-associated kinase (ACK) is an oncogenic nonreceptor tyrosine kinase associated with poor prognosis in several human cancers. ACK promotes proliferation, in part by contributing to the activation of Akt, the major effector of class 1A phosphoinositide 3-kinases (PI3Ks), which transduce signals *via* membrane phosphoinositol lipids. We now show that ACK also interacts with other key components of class 1A PI3K signaling, the PI3K regulatory subunits. We demonstrate ACK binds to all five PI3K regulatory subunit isoforms and directly phosphorylates p85α, p85β, p50α, and p55α on Tyr607 (or analogous residues). We found that phosphorylation of p85β promotes cell proliferation in HEK293T cells. We demonstrate that ACK interacts with p85α exclusively in nuclear-enriched cell fractions, where p85α phosphorylated at Tyr607 (pTyr607) also resides, and identify an interaction between pTyr607 and the N-terminal SH2 domain that supports dimerization of the regulatory subunits. We infer from this that ACK targets p110-independent p85 and further postulate that these regulatory subunit dimers undertake novel nuclear functions underpinning ACK activity. We conclude that these dimers represent a previously undescribed mode of regulation for the class1A PI3K regulatory subunits and potentially reveal additional avenues for therapeutic intervention.

Activated Cdc42-associated kinase (ACK) (encoded by *TNK2*) is a ubiquitously expressed nonreceptor tyrosine kinase first identified as an effector for the Rho family small G protein, Cdc42 (1). The N-terminal half of the 120 kDa ACK protein comprises a sterile alpha motif domain, nuclear export signal, tyrosine kinase domain, Src homology-3 (SH3) domain, and a Cdc42/Rac-interactive binding motif. The proline-rich C-terminal half of ACK includes a clathrin binding region, an epidermal growth factor (EGF) receptor-binding domain and a ubiquitin association domain (Fig. 1*A*).

**Figure 1:**
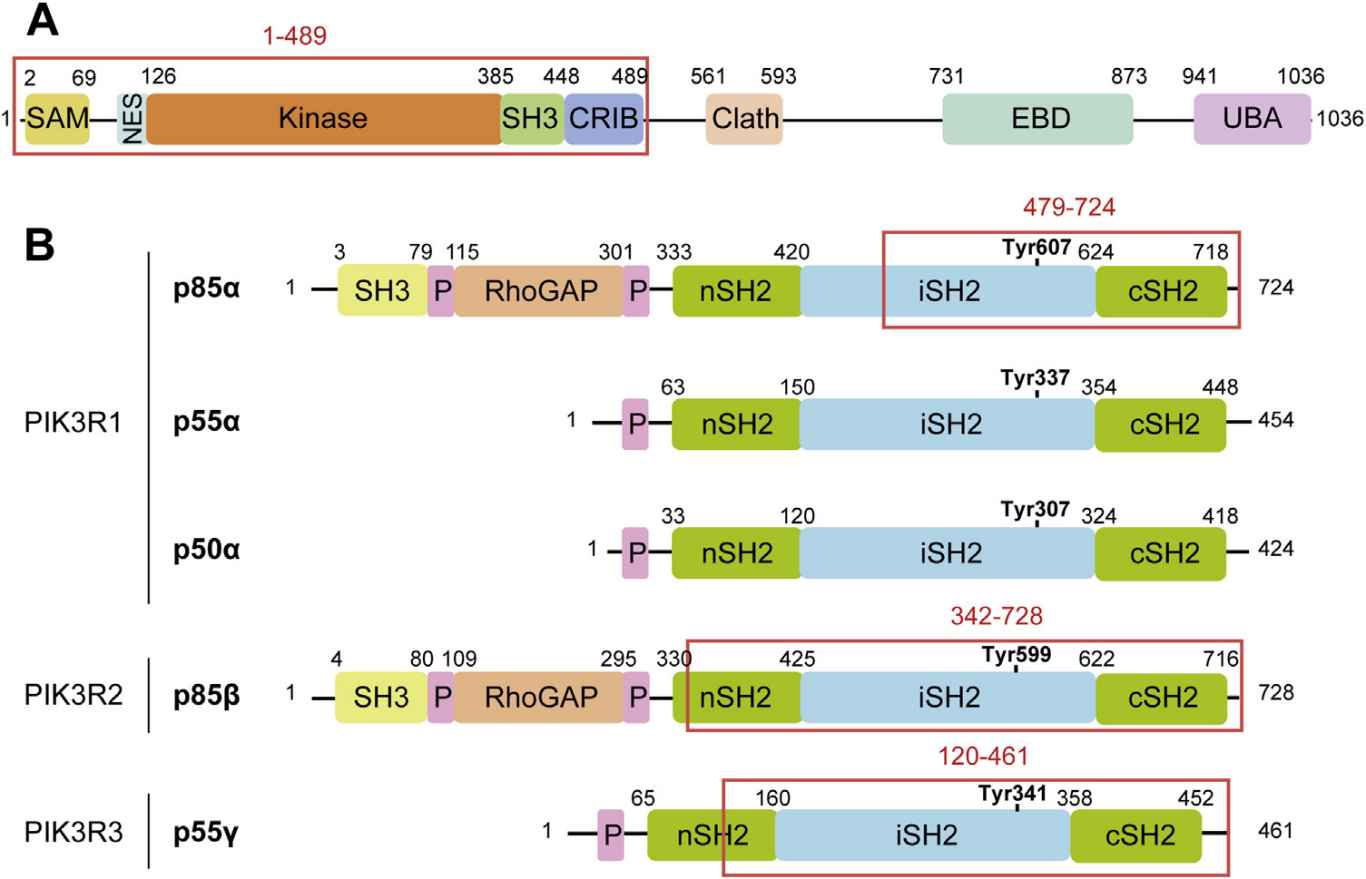
**Domain architecture of ACK and the p85 regulatory subunit with a summary of the yeast two hybrid results.***A*, domain architecture of ACK. SAM: sterile alpha motif domain, NES: nuclear export signal, SH3: Src homology-3 domain, CRIB: Cdc42/Rac-interactive binding motif, Clath: clathrin binding region, EBD: EGFR binding domain, UBA: ubiquitin association region. The region in the N-terminus of ACK used for the yeast-2-hybrid screen is indicated by the *red box*. *B*, domain architecture of class Ia PI3K regulatory subunits. SH3: Src homology-3 domain, P: proline-rich sequences, RhoGAP: region with sequence homology to Rho family GTPase-activating domains, SH2: Src homology-2 domain, nSH2: N-terminal SH2 domain, cSH2: C-terminal SH2 domain, iSH2: iSH2 region. Regions involved in interactions with ACK, as identified by the yeast-2-hybrid screen, are indicated by *red boxes*. The ACK phosphorylation site in p85α and the equivalent tyrosine residues in p50α, p55α, p85β, and p55γ are shown in *bold*. ACK, activated Cdc42-associated kinase.

In the past decade, ACK has emerged as a prospective therapeutic target in multiple human cancers. A genomic study, which sought to identify oncogenic ‘driver’ mutations within the protein kinase gene family in 210 diverse cancers, identified four such mutations in ACK (2), ranking ACK within the top ∼5% of kinases driving cancer. Amplification or mutation of *TNK2* is common in aggressive lung, ovarian, and hormone refractory prostate tumors, and increased levels of ACK mRNA correlate with poor patient prognosis (2, 3). The somatic mutations identified in ACK result in enhanced catalytic activity (2, 4). An oncogenic role for ACK is supported by studies showing activated ACK promotes the growth of prostate cancer xenografts (5). The regulatory mechanisms, signaling pathways, and future prospects to therapeutically target ACK in cancer have been recently reviewed (6) but ultimately, the successful development of therapeutic inhibitors to downregulate ACK signaling will rely on a detailed understanding of the pathways that lead to ACK activation and the signaling cascades that are activated downstream of ACK.

Relatively little is still known about the cellular substrates or interacting partners of ACK. In prostate cancer cell lines, ACK has been shown to phosphorylate the tumor suppressor Wwox, leading to its ubiquitination and degradation in the later stages of prostate cancer progression (5). ACK also phosphorylates histone H4, which leads to upregulation of androgen receptor (AR) (7) and in a co-ordinated manner, ACK phosphorylates the AR directly, activating its transcriptional activity (8). Both functions contribute to progression to the castration-resistant phase of prostate cancer.

PI3Ks catalyze the formation of the second messenger PIP_3_ from PIP_2_, which leads to the activation of pathways that promote cell proliferation and survival (9). Class 1A PI3Ks consist of a catalytic subunit (p110), of which there are three isoforms (p110α, β, and δ, encoded by *PIK3CA, PIK3CB,* and *PIK3CD* respectively), bound to a regulatory subunit (p85), which exists as five isoforms: p85α, p85β, p50α, p55α, and p55γ, encoded by three genes, *PIK3R1, PIK3R2,* and *PIK3R3* (10). The five regulatory isoforms have a similar domain organization in their C-terminal halves (Fig. 1*B*), comprising a proline-rich sequence and two Src homology-2 (SH2) domains, separated by a coiled–coiled domain termed the iSH2 (iSH2) region. The larger regulatory subunit isoforms, p85α and p85β, also possess additional functional domains at the N terminus, including a SH3 domain, another proline-rich sequence, and a RhoGAP domain (often referred to as the BH domain), which shows sequence homology to the GTPase-activating protein (GAP) domain of the breakpoint cluster region protein, Bcr (10).

The PI3K pathway is one of the most frequently dysregulated pathways in human cancers, with changes typically arising from activating mutations affecting the catalytic subunits: in fact, p110α is the most commonly mutated kinase in the human genome (11). Alterations in the regulatory subunits are less common. Most alterations identified are found in *PIK3R1* and result in decreased levels of the regulatory subunit or mutations that typically cluster within the iSH2 domain, disrupting the inhibitory contacts to p110 and resulting in PI3K pathway activation, suggesting that p85α has tumor suppressive properties (12). Alterations in *PIK3R2* and *PIK3R3* are even less common but often involve an increase in expression, suggesting an oncogenic role. Infrequent mutations have also been identified in *PIK3R2,* which seem to act in a similar manner to those in *PIK3R1*, resulting in PI3K activation. These data suggest that p85β may contribute to tumorigenesis by multiple mechanisms. Mutations in the regulatory subunits that do not manifest their effects through p110 have also been reported in endometrial and colorectal cancers, suggesting important p110-independent roles for p85 in cancer (13). The regulatory subunits exist in the cell in excess over the catalytic subunits, and these free subunits undertake crucial cellular functions independent of p110 (10, 14). Dimeric forms of p110-independent p85α have also been described that stabilize and increase PTEN phosphatase activity, which in turn counteracts PI3K activity by dephosphorylating PIP_3_, converting it back to PIP_2_ (15, 16, 17). Not surprisingly therefore, overactivation of PI3K signaling also results from mutations that reduce activity of the PTEN phosphatase. Mutations that activate the major downstream effector kinase, Akt, are also found in human cancers, albeit to a far lesser extent (18).

ACK directly contributes to the activation of the major PI3K effector, Akt. ACK phosphorylates Akt at Tyr176, which promotes its membrane localization and subsequent phosphorylation at Thr308/Ser473, resulting in its full activation (19). Here, we report that ACK also interacts with all five regulatory subunits of PI3K, phosphorylating four of them within the iSH2 domain at Tyr607 (or equivalent). Dimerization of free p85α has already been reported (15,16,17), but here, we describe a new mode of dimerization, in which the pTyr607 residue of one monomer interacts with the nSH2 domain of another, supporting formation of novel dimeric forms. This new mechanism of dimerization is also seen in the shorter isoforms, permitting multiple homodimer and heterodimer combinations. We demonstrate that the interaction between ACK and p85α occurs predominantly in nuclear-enriched cell fractions, where the resulting ACK-induced phosphodimers are also located. We show that mutation of the Tyr607 equivalent in p85β decreases proliferation in cells and propose that ACK modulates the PI3K response at multiple nodes including hereto unexplored functions of nuclear localized, C-terminal regulatory subunit dimers. These new dimers represent a previously undescribed mode of regulation for the class 1A PI3K regulatory subunits.

## Results

### Identification of the interaction between ACK and the PI3K regulatory subunits

A yeast-2-hybrid screen was performed to identify binding partners for ACK. A yeast strain, containing integrated GpYTH16-ACK(1–489) as bait, was screened against a pACT2-based human adult brain cDNA library, expressing prey proteins fused to the GAL4 activation domain. A human brain library was chosen as ACK was initially isolated from a human hippocampal expression library (1) and although ACK is ubiquitously expressed, it shows high levels in the brain (20). 169 β-galactosidase positive colonies were obtained, of which 114 remained after re-screening. Sequence analysis of the positive colonies revealed three isoforms of the PI3K regulatory subunit, p85α, p85β and p55γ, as the top hits for putative, novel ACK interacting proteins.

**Figure 2:**
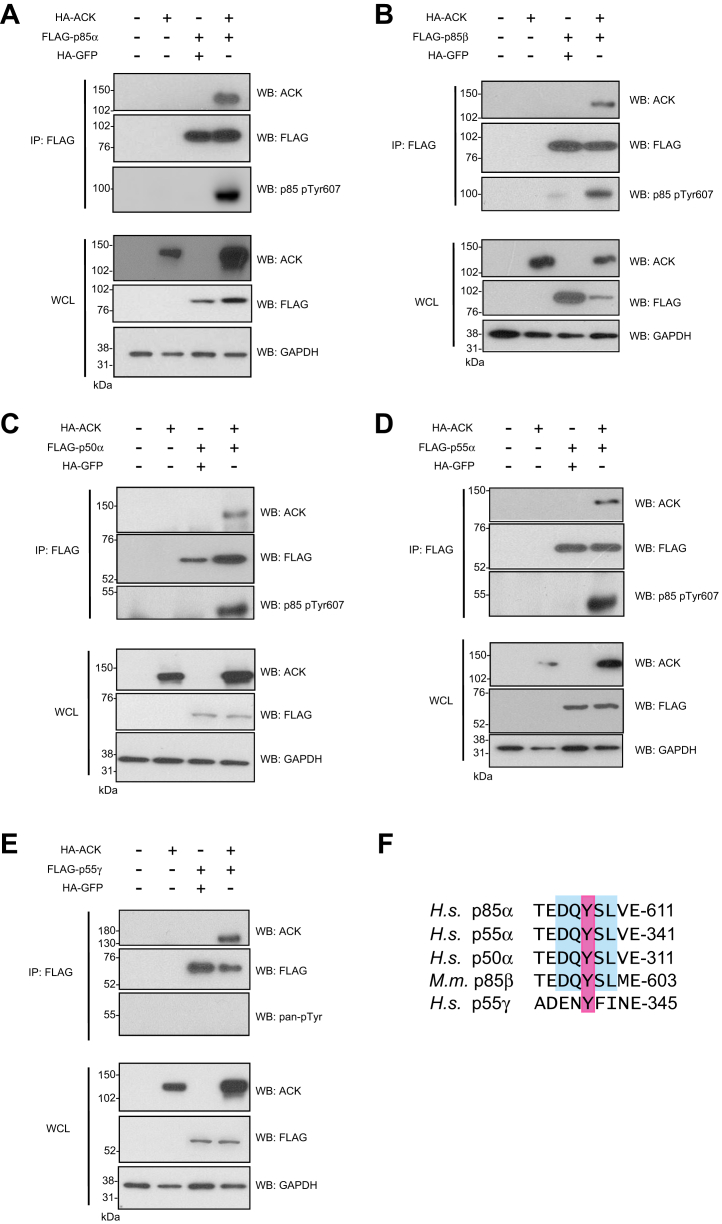
**Interaction and phosphorylation of full-length FLAG-tagged regulatory subunit isoforms by full length HA-ACK in HEK293T cells**. ACK was expressed alone or with FLAG-tagged PI3K regulatory subunit isoforms p85α (*A*), p85β (*B*), p50α (*C*), p55α (*D*), and p55γ (*E*). Expression of the recombinant proteins in whole cell lysate (WCL) is shown in the *bottom panels*. GAPDH is included as a loading control. PI3K regulatory subunit isoforms were immunoprecipitated from cell lysates using an anti-FLAG antibody, and the co-immunoprecipitation of ACK was assessed using an anti-ACK antibody (*top panels*). Phosphorylation of FLAG-tagged regulatory subunit isoforms was assessed by western blotting using an antibody raised against the p85 sequence (D-Q-Y_(p)_-S-L) and/or a pan anti-pTyr antibody. Results are representative of at least three independent experiments. *F*, amino acid sequence alignment surrounding Tyr607 or equivalent (*pink*) in all p85 isoforms. Sequence recognized by the anti-p85 (pTyr607) antibody is shown in *blue*. ACK, activated Cdc42-associated kinase.

The yeast-2-hybrid screen detected an interaction between the N-terminal half of ACK (1–489) and p85α (479–724), p85β (342–728) and p55γ (120–461) (Fig. 1), all of which included the C-terminal SH2 domain and most of the iSH2 region. The p85α splice variants, p50α and p55α, were not identified in the screen but were deemed likely interacting partners as they also harbor the C-terminal SH2 domain and iSH2 region. Co-expression of full-length ACK with all five full-length p85 isoforms individually in HEK293T cells, followed by immunoprecipitation, demonstrated that ACK interacted with all regulatory subunit isoforms (Fig. 2, *A*–*E*). We also observed an interaction between endogenous ACK and p85α in the prostate cancer cell line, LNCaP95 (Fig. S1).

### ACK phosphorylates p85α, p85β, p55α, and p50α at a conserved tyrosine within the iSH2 region

Several potential phosphotyrosine sites have been identified in the iSH2-cSH2 ACK-interacting region, and Tyr607 (p85α numbering), within the iSH2, has been reported to be a major phosphorylation site on p85α downstream of insulin receptor activation (21). Using an anti-p85 pTyr607 antibody, we found that p85α, p85β, p50α, and p55α were phosphorylated on Tyr607, or its equivalent, when they were co-expressed with ACK but not with a kinase-dead ACK variant (Figs. 2, *A*–*D* and S2). It was not possible to determine whether p55γ was phosphorylated on Tyr341 (equivalent to Tyr607 in p85α) when co-expressed with ACK using anti-p85 pTyr607, as p55γ does not possess the motif (D-Q-Y_(p)_-S-L) recognized by this antibody (Fig. 2*F*). However, the change in sequence context around this Tyr in p55γ also implied that it was unlikely to be an ACK substrate. Immunoblotting with a pan anti-pTyr antibody was used to assess whether p55γ showed increased tyrosine phosphorylation when co-expressed with ACK in cells. The lack of signal from the pan anti-pTyr antibody at 55 kDa (Figs. 2*E* and S2*E*) suggested that p55γ was not a substrate for ACK.

**Figure 3:**
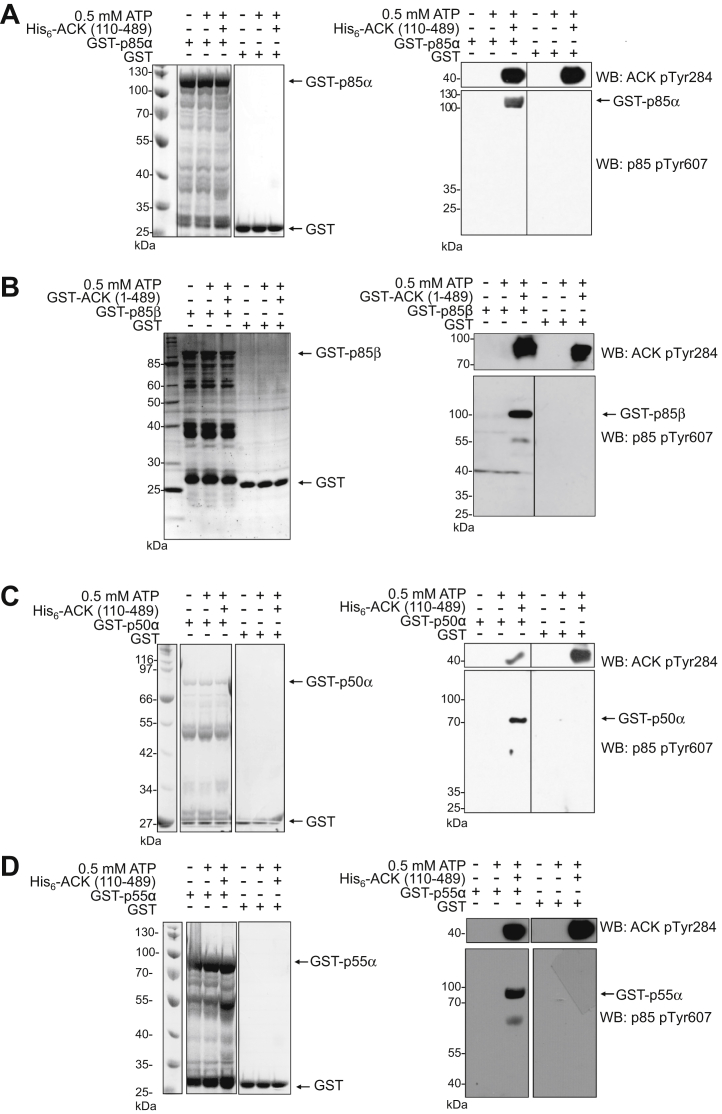
**Phosphorylation of the PI3K regulatory subunits *in vitro***. 30 μM Full-length GST-tagged regulatory subunits or GST control were incubated at 30 °C for 30 min with 0.3 μM His_6_-ACK (110–489) or GST-ACK (1–489). Reactions were analyzed by SDS-PAGE and Coomassie staining (*left panels*) and Western blotting using anti-p85 (pTyr607) and anti-ACK (pTyr284) antibodies (*right panels*). *A*, p85α is phosphorylated on Tyr607 by purified His_6_-ACK (110–189); *B*, p85β is phosphorylated on Tyr599 by purified GST-ACK (1–489); *C*, p50α is phosphorylated on Tyr307 by purified His_6_-ACK (110–189); and *D*, p55α is phosphorylated on Tyr337 by purified His_6_-ACK (110–189), *in vitro*. Note the samples in panels A and D were originally run on one gel, and the GST control samples have been duplicated for clarity. ACK, activated Cdc42-associated kinase.

To confirm that phosphorylation on Tyr607 by ACK was direct, *in vitro* kinase assays were performed using a purified, recombinant, active fragment of ACK. This was used to phosphorylate full-length regulatory subunit isoforms purified from *E. coli*. Incubation of GST-tagged p85α, p85β, p50α, or p55α with ACK resulted in their direct phosphorylation on Tyr607 (or equivalent), as assessed by immunoblotting (Fig. 3, *A*–*D* right panels).

To investigate whether ACK phosphorylates the regulatory subunits at additional tyrosine residues, the *in vitro* kinase assay using GST-p50α and ACK was repeated, the products resolved by gel electrophoresis and the phosphorylation site(s) mapped by mass spectrometry. Comparison of the mass spectra of samples with and without ACK detected phosphorylation of Tyr307 p50α (equivalent to Tyr607 in p85α) only when ACK was present (Fig. S3). No other tyrosine phosphorylation sites were identified, indicating that ACK phosphorylates p50α only at Tyr307 *in vitro*. To confirm that p55γ was not an ACK substrate, His_6_-MBP-p55γ was incubated with ACK, and phosphorylation sites were mapped by LC-MS/MS. No increase in p55γ tyrosine phosphorylation was detected following incubation with ACK, corroborating the results obtained in HEK293T cells.

### Phosphorylation of Tyr607 equivalent of p85β promotes cell proliferation

**Figure 4:**
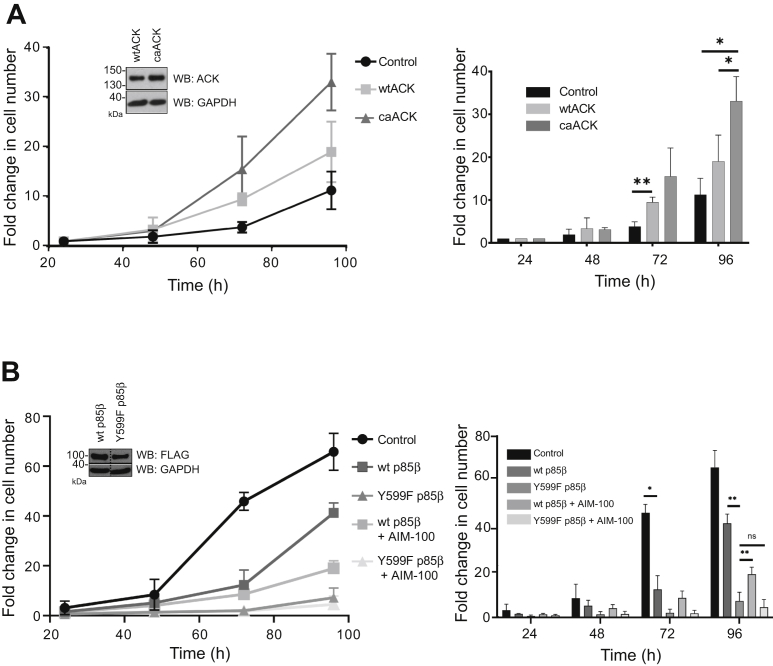
**Proliferation of HEK293T cells expressing ACK and p85β variants.***A*, proliferation of HEK293T cells stably expressing wtACK and caACK in 1% FBS. HEK293T cells stably expressing empty vector (control) or HA-tagged wtACK or caACK were seeded at a density of 3 × 10^4^ cells per well. Cells were plated in replicates of three, and live cell counts were obtained at the times indicated. Data are plotted as fold change over t = 0 (seeding density). Data from three independent experiments are shown. Error bars represent SD. Inset shows the relative levels of ACK in the WT and caACK cell lines by Western blot analysis using an anti-ACK antibody. GAPDH is included as a loading control. Data are represented on the right in a bar chart with group significance testing performed by a two-way ANOVA; ∗*p* <0.05; ∗∗*p* < 0.01. *B*, proliferation of HEK293T cells stably expressing empty vector (control), WT p85β, or Y599F p85β. HEK293T cells stably expressing inducible FLAG-tagged WT p85β or p85β Y599F were seeded at a density of 3 × 10^4^ cells per well and incubated at 37 °C for 16 h. All cells were then treated with 1 μg/ml doxycycline and incubated at 37 °C for the times indicated before live cell counts were taken. Data are plotted as fold change over the cell count at 24 h post doxycycline treatment. Certain cell lines were treated with the ACK inhibitor AIM-100 (8 μM) as indicated. Data from three independent experiments are shown. Error bars represent SD. Inset shows the relative levels of p85 in the WT and Y599F cell lines by Western blot analysis. Data are represented on the right in a bar chart with group significance testing performed by a two-way ANOVA; ∗*p* <0.05; ∗∗*p* < 0.01. ACK, activated Cdc42-associated kinase; caACK, constitutively active ACK.

The ACK target site is located within the iSH2 region of the regulatory subunits (Fig. 1*B*). The iSH2 region is known to provide important regulatory contacts with the p110 catalytic subunit (22), and insulin receptor activation results in phosphorylation at this site *in vivo* (21). To investigate the effects of this tyrosine on proliferation, HEK293T clonal cell lines were generated, stably expressing wildtype (WT) ACK, constitutively active ACK (caACK) (5), WT p85β or a nonphosphorylatable p85β mutant, Y599F (equivalent to Y607F). Cell lines with equivalent ACK expression levels (see insets, Fig. 4) were tested for comparative rates of proliferation. Proliferation assays confirmed that the introduction of either WT or caACK into HEK293T cells conferred a proliferative advantage over a polyclonal control cell line (Figs. 4*A* and S4*A*). The proliferation of caACK expressing cells was significantly faster than wtACK cells over 96 h, suggesting that the kinase activity of ACK was the driving force in stimulating cell proliferation. In contrast, cells expressing WT p85β grew at a slower rate than control cells (Figs. 4*B* and S4*B*) demonstrating that p85β inhibits proliferation. This is in agreement with previous studies indicating that increased levels of p85 negatively regulate PI3K activity (23, 24). Cells expressing Y599F p85β however proliferated at an even slower rate suggesting that the inhibitory effect of p85β is counteracted by phosphorylation of Tyr599. The effects of the ACK-specific inhibitor AIM-100 (25) were also tested. Cells expressing WT p85β grew at a reduced rate in the presence of AIM-100 (Figs. 4*B* and S4*B*), suggesting that phosphorylation at Tyr599 by endogenous ACK counteracts some of the antiproliferative effects of p85β, while AIM-100 did not affect cells expressing Y599F p85β. Overall, these data suggest that ACK stimulates cell proliferation by phosphorylating p85β at Tyr599.

### Identification of the pTyr607–nSH2 interaction

The ACK substrate tyrosine resides in an unstructured region of the p85 iSH2 domain, which is missing in the crystal structure of the p110α/p85 niSH2 heterodimer (26). As a result, the accessibility of this residue in the heterodimer cannot currently be confirmed. However, as the nSH2-iSH2 region is known to form important regulatory contacts to the catalytic subunit, the likelihood that Tyr607 (or equivalent) is inaccessible in the PI3K heterodimer led us to speculate that ACK targets the pool of p110-independent p85. The iSH2 domain, containing the ACK target site, is flanked by the N-terminal (nSH2) and C-terminal (cSH2) SH2 domains (Fig. 1*B*). The ACK target tyrosine is conserved among regulatory isoforms (Fig. 2*F*) and conforms to the p85 SH2 binding consensus sequence (pYXXM) in p85β (27). Therefore, we hypothesized that there might be an interaction between one of the flanking SH2 domains and the phosphotyrosine produced by ACK. This interaction could modulate the conformation, stability and hence cellular activity of p110-independent p85, and potentially underpin the proliferative potential of pTyr599 p85β.

**Figure 5:**
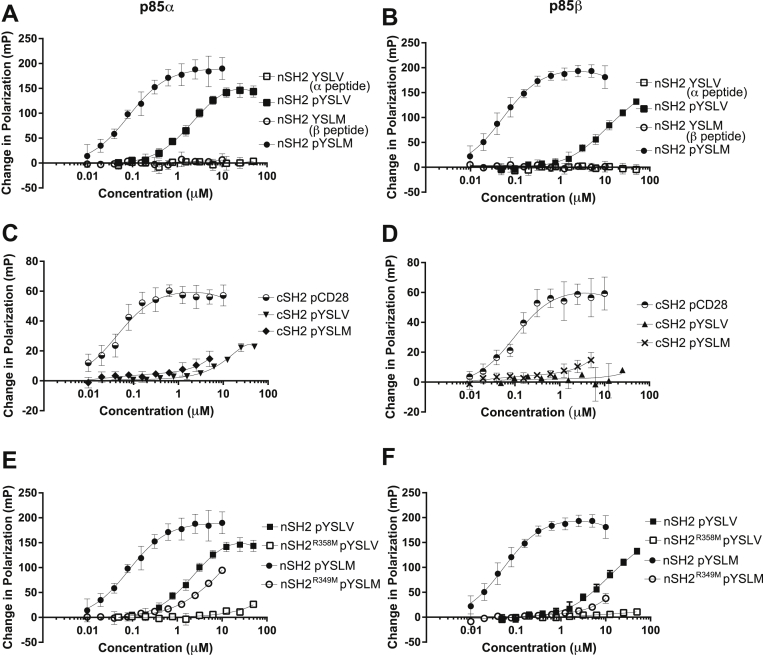
**Binding of the p85 SH2 domains to phosphopeptides.** Purified nSH2 and cSH2 domains from p85α and p85β were analyzed for binding to fluorescein-labeled phosphopeptides based on the sequences surrounding Tyr607 in p85α and p85β, in fluorescence polarization assays. Data from three experiments are shown and the error bars represent the SEM. *A*, binding of the nSH2 domain of p85α to phosphorylated and nonphosphorylated peptides based on p85α and p85β. *B*, binding of the nSH2 domain of p85β to phosphorylated and nonphosphorylated peptides based on p85α and p85β. *C*, binding of the cSH2 domain of p85α to phosphorylated peptides based on p85α, p85β and a control peptide from CD28. *D*, binding of the cSH2 domain of p85β to phosphorylated peptides based on p85α, p85β and a control peptide from CD28. *E*, binding of the WT nSH2 domain of p85α and the R358M variant to phosphorylated peptides based on p85α and p85β. *F*, binding of the WT nSH2 domain of p85β and the R349M variant to phosphorylated peptides based on p85α and p85β. cSH2, C-terminal SH2 domain; nSH2, N-terminal SH2 domain.

To test this hypothesis, we expressed and purified each SH2 domain from p85α and p85β in *E. coli.* We then tested their ability to interact with peptides containing the target tyrosine and surrounding sequence from p85α and p85β using fluorescence polarization. The nSH2 domains of both p85α and p85β interacted with the p85α (pYSLV) peptide with low micromolar affinity and bound with higher affinity (low nanomolar) to a p85β (pYSLM) peptide, with the binding dependent on phosphorylation of the Tyr (Fig. 5, *A* and *B* and Table 1). In contrast, very little binding was observed between the peptides and the cSH2 domains of either p85α or p85β (Fig. 5, *C* and *D*). To confirm that the bacterially expressed cSH2 domains were properly folded, binding to a known target peptide from CD28 was tested (Fig. 5, *C* and *D*). Binding affinities similar to those previously reported were observed, indicating that both cSH2 domains were capable of binding phosphopeptides (27). Thus, pTyr607 (or equivalent) supports an interaction with both p85α and p85β nSH2 domains, and the p85β peptide binds with a higher affinity.

**Table 1:** Equilibrium binding constants for p85 SH2 domains and phosphorylated peptides

*K*_d_ (nM)^a^	pYSLV (α peptide)	pYSLM (β peptide)	pCD28 peptide
p85α nSH2	2400 ± 315	82 ± 16	−
p85β nSH2	9000 ± 282	52 ± 8	−
p85α cSH2	84,000	656 ± 424	43 ± 10
p85β cSH2	ND^b^	ND	100 ± 27
p85α nSH2 R358M	ND	2900 ± 1600	−
p85β nSH2 R349M	ND	ND	−

a Standard error where n = 3.

b ND (no binding) denotes data that could not be fitted to the binding isotherm.

We then tested the binding mode of the p85 nSH2 domains. An arginine residue is evolutionarily conserved in SH2 domains and forms a hydrogen bond with the phosphate group of the phosphotyrosine residue on binding targets (28). Mutation of this arginine to methionine in p85α completely abolishes phosphopeptide binding without disrupting the characteristic SH2 fold (27). Site-directed mutagenesis was used to generate SH2 mutants p85α R358M and p85β R349M, and the variant domains were analyzed for peptide binding. These nSH2 null mutations reduced binding to the phosphopeptide sequence by several orders of magnitude, indicating that the WT nSH2-pTyr607 binding mechanism is likely to be similar to other canonical p85 SH2–peptide interactions (Fig. 5, *E* and *F*).

### The pTyr607–nSH2 interaction occurs in trans and promotes formation of novel regulatory subunit dimers

Theoretically, the interaction between the nSH2 and pTyr607 of the regulatory subunits could occur either *in cis* in a monomeric protein or *in trans* in a dimer. To investigate whether the nSH2–pTyr607 interaction could support dimer formation, we co-expressed either FLAG-p50α with V5-p85β or FLAG-p85α with V5-p85β. We chose to study p85β because peptides from this isoform had the highest affinity binding to the nSH2 domains tested. The longer p85α regulatory isoforms are already known to dimerize *via* N-terminal domain interactions: SH3:PR1 and BH:BH. To distinguish between dimers formed through interactions located in the N-terminal or C-terminal half of the molecules, we utilized the p50α isoform, which lacks the N-terminal SH3, polyproline, and RhoGAP/BH regions.

**Figure 6:**
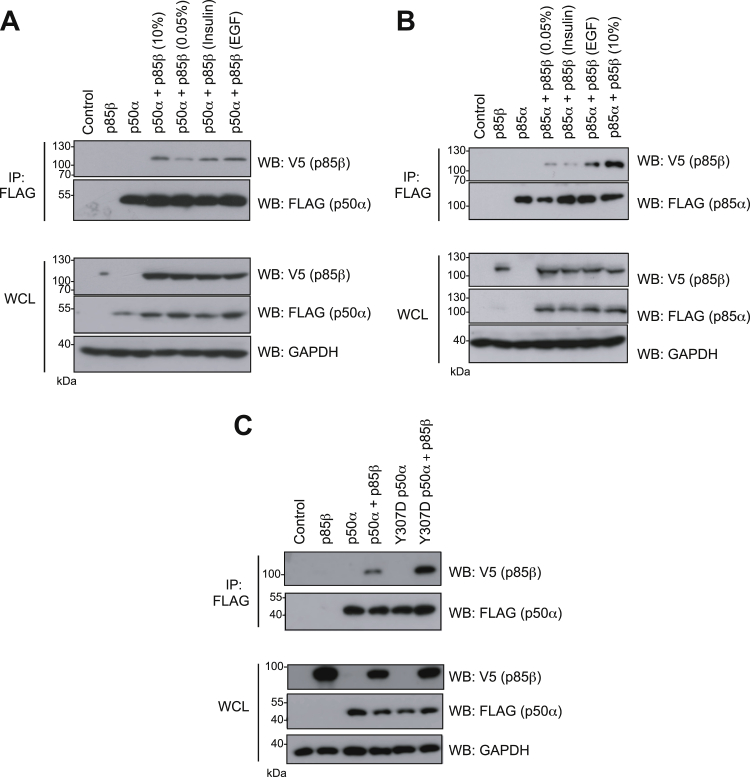
**Dimerization of the regulatory subunits.** HEK293T cells were transfected with control (GFP), FLAG-p50α (*A*) or FLAG-p85α (*B*) alone or with V5-p85β. Expression of the proteins in whole cell lysates (WCLs) is shown in the bottom panels. As indicated, cells were cultured in high serum (10% serum) or serum starved (0.05% serum) for 16 h and prior to lysis were either left untreated or stimulated with either EGF (100 ng/ml for 5 min) or insulin (100 μg/ml for 90 min). Cells were harvested 40 h after transfection and subject to immunoprecipitation using anti-FLAG antibodies. Co-precipitation of V5-p85β was assessed by Western blotting of immunoprecipitated samples using anti-V5 antibodies (*top panels*). *C*, WT p85β, WT p50α, and Y307D p50α (equivalent to Tyr607 in p85α) were expressed alone or co-expressed in HEK293T cells cultured in low serum. Samples of WCLs were analyzed by Western blotting using the antibodies indicated and are shown in lower panels. GAPDH is included as a loading control. FLAG-tagged p50α variants were immunoprecipitated from cell lysates using an anti-FLAG antibody, and the co-immunoprecipitation of p85β was assessed using an anti-V5 antibody (*top panels*). Results are representative of at least three independent experiments.

Proteins were co-expressed in HEK293T cells cultured in the presence of either 10% serum or in low serum (0.05%) followed by treatment with EGF (a known stimulator of ACK (29)) or insulin (Tyr607 phosphorylation has been detected downstream of insulin stimulation (21)). p50α interacted with p85β under all conditions but increased complex formation was observed in high serum and in response to insulin (Fig. 6*A*), suggesting that dimerization is induced by growth factor signaling. Dimerization in this case must be occurring solely *via* C-terminal interactions. p85α was also seen to interact with p85β (Fig. 6*B*). Again, lower levels of complex were seen in low serum, with an increase in 10% serum or following EGF stimulation (Fig. 6*B*), although this interaction was not enhanced by insulin treatment. To confirm that the interactions observed were mediated by the pTyr607–nSH2 interaction, we investigated the interaction between p85β and a p50α phosphomimic mutant (Y307D, equivalent to Y607D in p85α) (Fig. 6*C*). Although substitution of tyrosine with aspartate is not always an ideal phosphotyrosine mimic, it is effective in many cases and this approach has been utilized successfully previously for analying p85 SH2 domain–phosphotyrosine interactions (30). Whereas in low serum conditions, WT p50α–p85β complexes were detected at low levels, as previously observed, significantly more Y307D p50α–p85β dimer was seen, suggesting that phosphorylation at Tyr307 underpins this interaction.

These data demonstrate that the C-terminal regions, and specifically the nSH2–pTyr607 interaction, support formation of a novel dimeric configuration of the regulatory subunits in response to growth signals.

### ACK interacts with p85α in nuclear-enriched cell fractions

We have demonstrated that ACK phosphorylates the PI3K regulatory subunits on Tyr607 and that this phosphorylation triggers dimerization of the PI3K regulatory subunits. Functional investigation of p110-independent p85 is an emerging field and has revealed roles in cellular stress response pathways and in the nucleus (10, 31, 32). While PI3K and ACK are primarily activated by receptor tyrosine kinases at the plasma membrane, nuclear translocation of ACK (8, 33) and the p85α regulatory subunit (31, 34) have also been documented.

**Figure 7:**
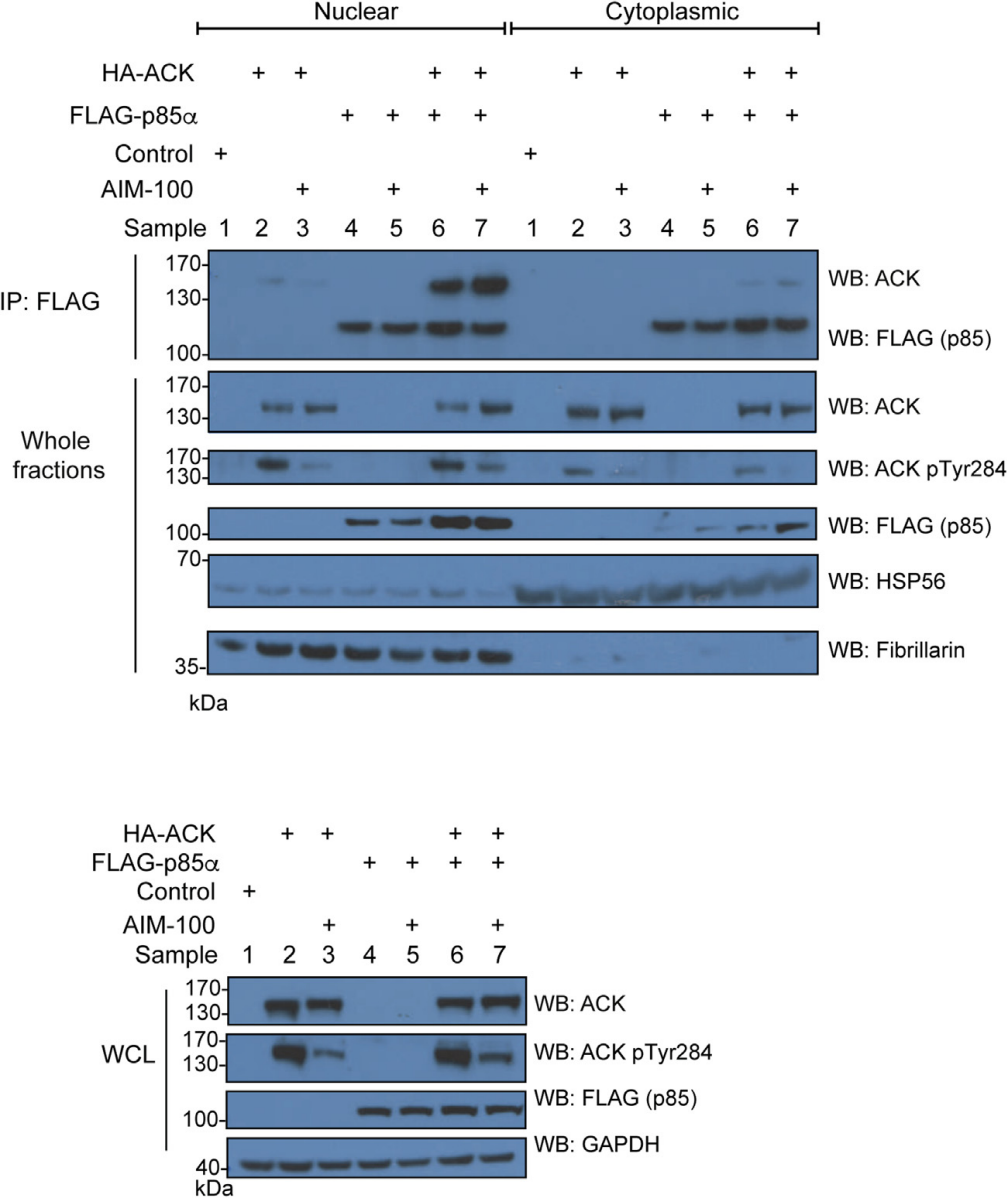
**Subcellular localization of the ACK–p85**α **complex.** ACK and WT p85α were expressed alone and together in HEK293T cells. Where indicated, cells were treated with 8 μM AIM-100. Fractionation was judged using anti-HSP56 to indicate cytoplasmic extracts and anti-fibrillarin to indicate nuclear-enriched fractions. Nuclear and cytoplasmic-enriched fractions were normalized using the Bradford assay, and FLAG-p85α was immunoprecipitated from fractions containing equal total protein using an anti-FLAG antibody. Top panel: Immunoprecipitation of FLAG-p85α and co-immunoprecipitation of HA-ACK was assessed by Western blotting using anti-FLAG and anti-HA antibodies. Bottom panel: Expression of FLAG-p85α and HA-ACK along with active ACK levels was confirmed by Western blotting of whole cell lysate (WCL) samples collected prior to cell fractionation. ACK, activated Cdc42-associated kinase.

To determine the subcellular location of ACK–p85α complexes, HEK293T cells were transiently transfected with ACK and p85α expression constructs, separated into cytoplasmic and nuclear-enriched fractions, and the p85α immunoprecipitated. The interaction between p85α and ACK was detected almost exclusively in nuclear-enriched fractions (Fig. 7), suggesting that phosphorylation of p85 by ACK also occurs in the nucleus. Consistent with this, p85α pTyr607 was almost entirely located in the nuclear fractions (Fig. S5). Suppression of ACK activity with AIM-100 reduced active ACK levels, as assessed by blotting for pTyr284 ACK, but did not block p85α–ACK complex formation in the nucleus, suggesting that ACK kinase activity is not required for the interaction with the PI3K regulatory subunits (Fig. 7).

### The pTyr607–nSH2 regulatory subunit dimers reside in the nucleus

**Figure 8:**
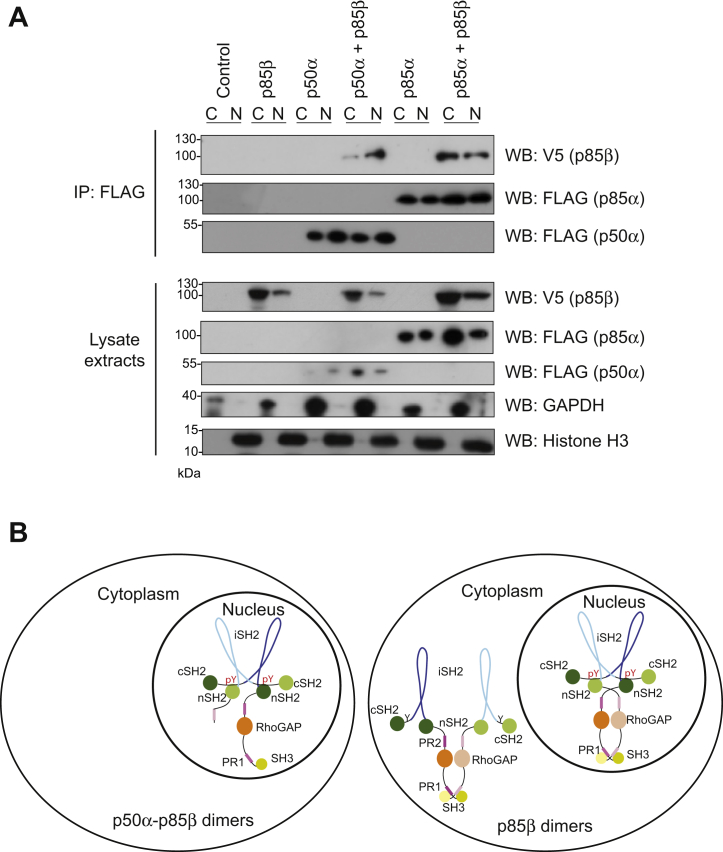
**Subcellular localization of the nSH2–pTyr607 regulatory subunit dimers.***A*, HEK293T cells were transfected with a control vector (GFP), FLAG-tagged WT p85β, WT p85α, and WT p50α either alone or together as indicated. Cells were lysed and separated into cytoplasmic and nuclear-enriched fractions. Fractions were analyzed by Western blotting using the antibodies indicated (*lower panels*). Fractionation was judged using anti-GAPDH to indicate cytoplasmic extracts and anti-Histone H3 to indicate nuclear-enriched fractions. FLAG-tagged p50α or p85α were immunoprecipitated from cell lysates using an anti-FLAG antibody, and the co-immunoprecipitation of p85β was assessed using an anti-V5 antibody (*top panels*). Results are representative of at least three independent experiments. *B*, schematic to show potential dimer configurations in the nucleus only (p50α–p85β, *left*) or in the cytoplasm and nucleus (p85β–p85β, *right*). In each cartoon, one monomer is in pale tones, and the other in darker tones, with the domains being colored as in Figure 1*B*.

Since both the ACK–p85α interaction and p85α pTyr607 occur almost exclusively in the nuclear-enriched fractions (Figs. 7 and S5), we investigated the subcellular location of regulatory subunit dimers. The regulatory subunits were co-expressed, and the cells fractionated before immunoprecipitation with anti-FLAG antibodies. p50α–p85β dimers were found mainly in the nucleus, whereas p85α–p85β dimers were located in both the cytoplasm and the nucleus (Fig. 8*A*). We interpret these data to indicate that p50α-p85β dimers, which must be C-terminal dimers, are found in the nucleus, whereas p85α–p85β, which are a mixture of both types of dimer (N-terminal dimers and those that also involve the C-terminal interaction), are found in both cellular compartments (Fig. 8*B*). The nuclear subset is likely to comprise C-terminal dimers, mediated by the pTyr607–nSH2 interaction, while the cytoplasmic will be in the N-terminal dimer configuration. While the nuclear roles of the pTyr607–nSH2 p85 dimers remain unknown, we predict that they may drive cell proliferation.

## Discussion

**Figure 10:**
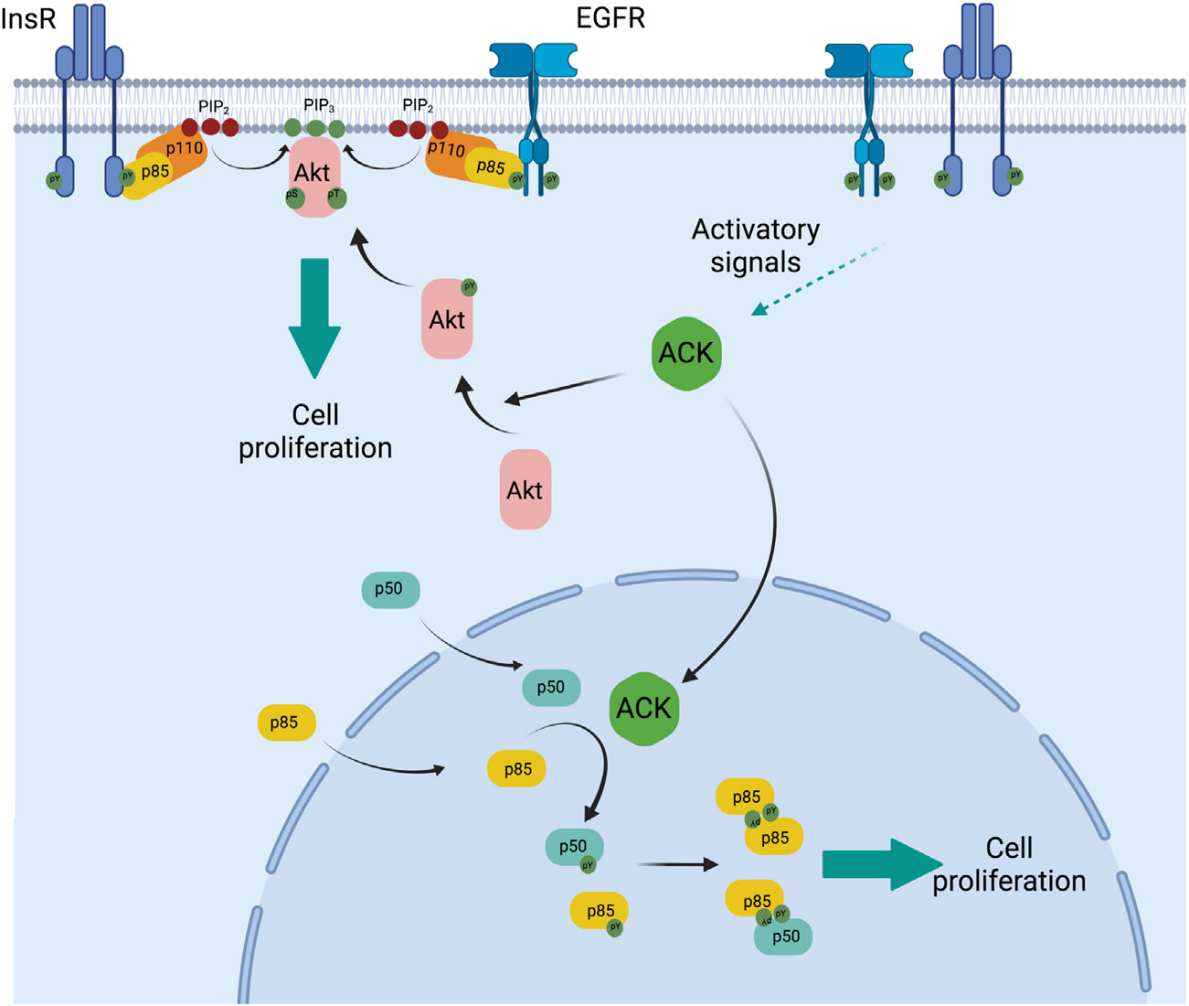
**ACK regulation of the PI3K signaling pathway.** Cartoon summarizing the points of influence of ACK on components of the PI3-kinase signaling pathway. Phosphorylation sites are shown as *green circles*. ACK, Activated Cdc42-associated Kinase; Akt, Ak strain transforming protein; EGFR, EGF receptor; InsR, insulin receptor; p50, short isoform of the PI3K regulatory subunit; p85, long isoform of the PI3K regulatory subunit; p110, PI3k catalytic subunit; PIP_2_: phosphatidylinositol 4,5-bisphosphate, PIP_3_: phosphatidylinositol (3, 4, 5)-trisphosphate. This figure was created in Biorender.

We have identified the five isoforms of the class 1A PI3K regulatory subunits as novel ACK binding partners. The majority of our data were derived from exogenously expressed proteins, although we also observed the endogenous ACK–p85α interaction in LNCaP95 cells. Interestingly, the p55γ isoform was also recently identified as a binding partner for ACK in the Human Reference Interactome map study (35), further supporting our findings. We have also shown that p85α, p85β, p55α, and p50α are direct substrates for ACK kinase activity. These isoforms are phosphorylated by ACK at a conserved tyrosine residue in the iSH2 domain of the protein (equivalent to Tyr607 in p85α). We have shown that the phosphorylation status of this residue in the p85β isoform affects cell proliferation in culture. We have discovered that phosphorylation of the regulatory subunits at Tyr607 (or equivalent) induces their dimerization, mediated by an interaction involving the nSH2 domain. These phosphorylation-induced dimers are localized in nuclear-enriched cell fractions, in accordance with the nuclear localization of the ACK–p85 complexes and resulting pTyr607 p85α. We propose that ACK may drive cell proliferation through hitherto unexplored but crucial functions of these novel PI3K regulatory subunit dimers (Fig. 10).

There is a superabundance of p85 subunits over the catalytic subunits in cells and the excess regulatory subunits exist independently of p110 (14, 23). Free p85α can form homodimers mediated by interactions between N-terminal protein domains: a *trans* interaction has been identified between the SH3 domain and its juxtaposed proline-rich motif (PR1) (17, 36) and the RhoGAP (BH) domain has also been shown to mediate homodimerization (36). Both of these interactions are relatively weak and it has been proposed that multiple sites of interaction are necessary to produce a stable p85 homodimer, allowing multiple levels of co-ordinated regulation as is common in signaling systems (16, 36).

In contrast, the pTyr607–nSH2 interaction we have identified provides a novel mechanism mediating dimerization of the PI3K regulatory subunits *via* their C-terminal regions. The binding mode is likely to be similar to that already described for the p85 nSH2 domain with other phosphopeptides (27), as shown by reduced binding by the R358M SH2–null mutant. The interaction is high affinity and therefore sufficient to support dimerization alone, in the absence of other contributing interactions, as p50–p85 heterodimers could be observed (Figs. 6 and 8). Unlike the SH3–PR1–mediated and BH-mediated dimerization modes, both of which involve N-terminal regions of the protein, the pTyr607-nSH2 interaction allows dimerization of the shorter isoforms p50α and likely p55α. Our data demonstrate that heterodimerization is supported between p85β and both p50α and p85α, but could potentially occur between any of the four phosphorylated regulatory subunit isoforms, as the nSH2 domains from p85α and p85β bind to phosphopeptides representing sequences from both p85α and p85β. The nSH2 and peptide sequences for p85α are identical in the shorter variants p55α and p50α, implying that any combination of isoforms could form heterodimers. Our affinity data indicate preference for binding to the p85β peptide in both cases, which is supported by the p85β peptide conforming to the consensus sequence for a p85 SH2 domain binding target (pYXXM). This suggests that α isoforms should preferentially heterodimerize with p85β, while p85β would preferentially form homodimers. Importantly, this new mechanism of dimerization is regulated by phosphorylation at Tyr607.

**Figure 9:**
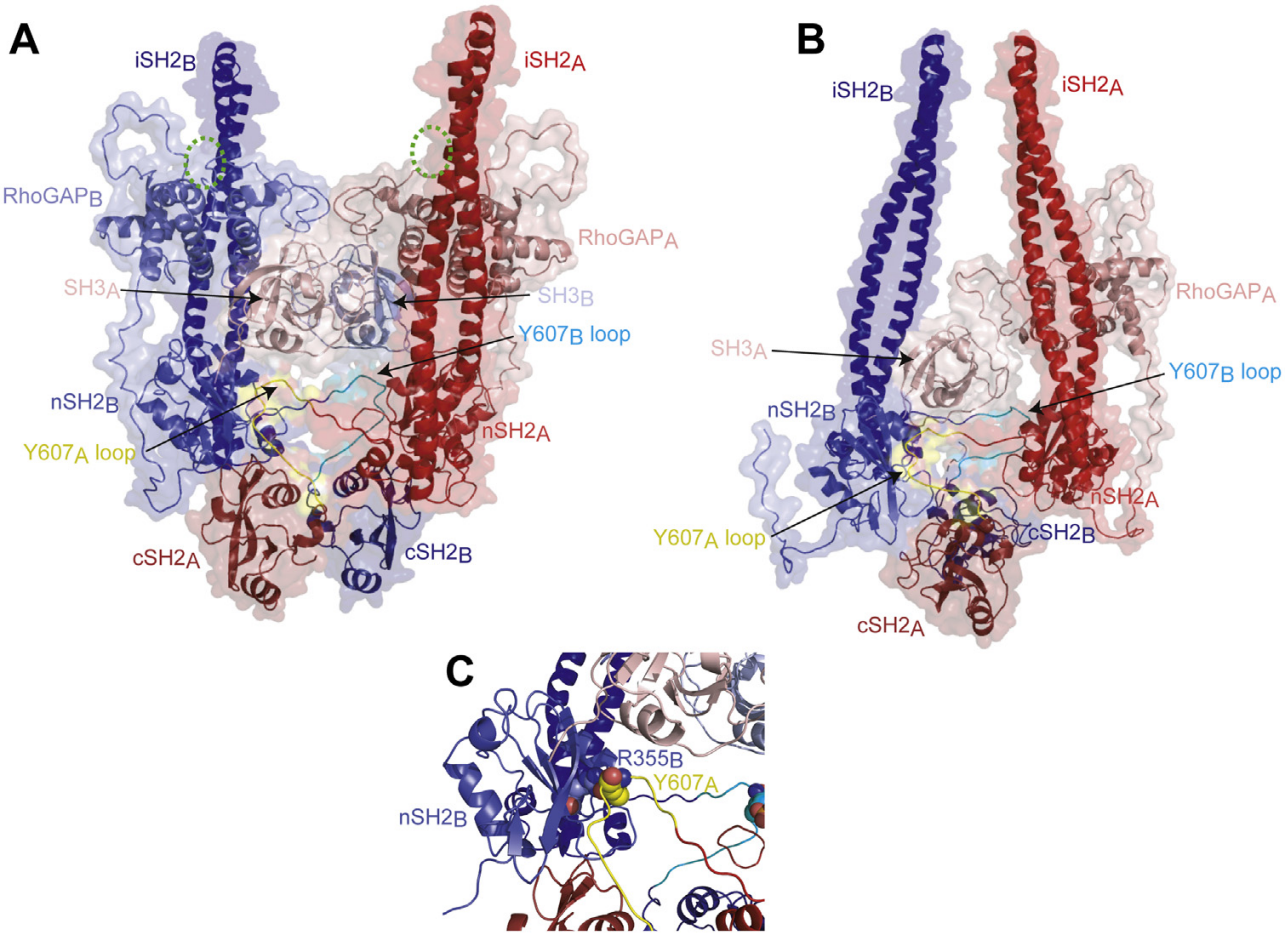
**Structural model of the novel nSH2–pTyr607–mediated dimers.***A*, the potential p85β homodimer. The two chains of the dimer are shown in *red* (monomer A) and *blue* (monomer B), with pale shades at the N terminus and darker shades at the C terminus. The G protein binding site of the BH domain is indicated by a *green dotted circle*. The loops that contain pTyr607 are colored yellow (monomer A) and cyan (monomer). *B*, the potential p85α–p50α heterodimer, colors as above except that p50α is *blue* and p85α is *red*. *C*, close up view showing the details of the intermolecular interaction between pTyr607 (*yellow*) and the nSH2 (*blue*) of the p85α–p85α dimer. The side chains of Tyr607 in monomer A and the interacting Arg355 in the monomer B are shown as spheres, with carbons in *yellow* (monomer A) or *pale blue* (monomer B), oxygen in *red*, nitrogen in *dark blue,* and phosphate in *orange* (not visible).

Using the HADDOCK docking server, we constructed a model of how these dimers could form, using the AlphaFold coordinates of monomeric human p85β for each monomer but allowing flexibility for the linkers between the domains. The models were derived using intermolecular restraints between nSH2 and phosphopeptide, between the SH3 domain and the first Pro-rich region (17) and between the RhoGAP domains (36). The top-scoring model is compatible with all three dimerization interactions and given that all three known interactions can be satisfied in one model, they could occur simultaneously (Fig. 9*A*). It is likely however, that the addition of the nSH2–pTyr607 interaction to a dimer formed through N-terminal interactions leads to reorientation of the dimer. Importantly, a p85 dimer where the nSH2 domain is bound to pTyr607 *in trans* is unlikely to retain the ability to bind to the p110 catalytic subunits, as multiple contacts to the p110 subunit occur within the nSH2–iSH2 region of the regulatory subunits. A p50-p85 model was also constructed, which shows a reorientation of the domains (Fig. 9*B*) as the N-terminal interactions no longer need to be accommodated. Although this may suggest that the two dimers are distinct, it is more likely that larger-scale domain rearrangements take place to allow C-terminal dimer formation and that the domains orientations are not fixed.

p110-independent p85 is still an emerging area of research but to date the roles reported for free p85 are those of adaptor proteins directing the cellular localization of key signaling molecules (10). Free p85 plays roles in various cell stress response pathways, regulating p53, XBP-1, and BRD7 (31, 32, 34, 37, 38). Typically, the adaptor functions of free p85 rely on the N-terminal domains of the protein. The RhoGAP/BH domain resides in the N-terminal region and binds both Rac1 and Cdc42, although GAP activity toward either small G protein, if it exists at all, is very low (39). The RhoGAP/BH domain has also been shown to be a GAP for Rab proteins, including Rab5, albeit with equally low activity, and to therefore play a role in receptor endocytosis (39, 40). The N-terminal regions of p85 are also involved in the regulation of PTEN activity, counteracting PI3K signaling (41). The SH3 domain of the p85 subunits interacts with many target proteins, which, in different contexts can confer mechanisms that activate PI3K independent of the SH2 domains, inhibit PI3K, regulate endocytosis, or regulate the p85 protein itself (42). The C-terminal dimers we describe here would leave the the RhoGAP domain available to engage substrates (Fig. 9*A*). Interactions involving the other N-terminal domains could also be functionally altered in this new dimeric configuration. However, it remains to be determined which interactions and cellular processes the pTyr607–nSH2 regulatory subunit dimers can support and therefore their function(s).

ACK has been shown to shuttle between the cytoplasm and the nucleus in a Cdc42-dependent manner (33). Once in the nucleus, it has a number of important roles in regulating transcription, *e.g.,* by influencing the AR and FoxO transcription factors (7, 43). Similarly, many of the roles of free p85 are in the nucleus and revolve around regulation of transcription of genes that control cell proliferation or senescence, alongside protection from different forms of cell stress. Our data support a model where ACK and p85 primarily interact in the nucleus, and subsequently, the regulatory subunit dimers that form also appear to exist predominantly in the nuclear-enriched cell fractions, suggesting a novel nuclear function for the new dimer configuration described here (Fig. 10).

The work presented here, along with a growing body of literature describing the cellular effects of ACK, indicates that ACK regulates PI3K signaling at multiple nodes. ACK has the ability to drive proproliferative pathways *via* Akt(19), allowing it to direct p85 to perform its other vital roles in the nucleus. We have identified a new form of phosphorylation-dependent, high-affinity PI3K regulatory subunit dimers, which appear to form exclusively in the nucleus and potentially undertake proproliferative roles.

## Experimental procedures

### Yeast-2-hybrid screen

ACK (1–489) was cloned into pENTR/D-TOPO and then transferred into destination vectors GpYTH9 and GpYTH16, using Gateway recombination. This produced recombinant yeast expression bait constructs with the ACK coding region in frame with the DNA-binding domain of Gal4. The GpYTH16 construct was used to assess transactivation in *S. cerevisiae* using a β-galactosidase assay, while the GpYTH9 construct was linearized with *Xba*1 and integrated into the yeast strain Y190::ADE2 (MATa, ura3-52, his3-Δ200, lys2-801, trp1-901, leu2-3, 112, gal4Δ, gal80Δ, cyh^r^2, LYS2::GAL1_UAS_-HIS3_TATA_-HIS3, MEL1 URA3::GAL _UAS_-GAL1_TATA_-lacZ, GAL2 _UAS_-GAL2 _TATA_-ADE2) for screening. Transformants were selected on media lacking tryptophan. The resulting strain was transformed with a Matchmaker Human Fetal Brain cDNA library (Clontech), where prey cDNAs are cloned in frame with the activation domain of Gal4 in pACT2. Transformants were selected on media lacking leucine but containing X-gal (5-Bromo-4-Chloro-3-Indolyl β-D-Galactopyranoside). Large blue colonies were picked onto fresh selective plates and then reassayed for β-galactosidase activity. Yeast colonies were transferred onto Whatman No.54 filter papers and lysed by freeze thawing. Each filter was then immersed in 60 mM NaH_2_PO_4_, 40 mM NaH_2_PO_4_, 10 mM KCl, 0.1 mM MgSO_4_, 40 mM β-mercaptoethanol, 1 mg/ml X-gal, pH 7.0, and incubated at 37 °C for 4 h. Filters were air-dried and checked for blue coloration. Library plasmids isolated from the β-galactosidase-expressing Leu^+^ yeast were recovered and their sequences analyzed.

### Plasmids and site-directed mutagenesis

pXJ-HA-ACK was a kind gift from Prof Ed Manser (ICMB, Singapore). Full-length human p85α, p50α, p55α, and p55γ and mouse p85β cDNA (IMAGE) were amplified by PCR, cloned into pENTR/D-TOPO, and then transferred into the mammalian expression vector pDEST12.2-FLAG and the bacterial expression vectors pDEST15 and pETG-41A using Gateway technology. p85β cDNA was also cloned into the lentiviral vector, pSLIK-hygro. The coding sequences of WT and caACK were digested from pXJ-HA constructs *Eco*RI/*Xho*I and ligated into the retroviral expression vector, pBABE-puro. Site-directed mutagenesis was performed using a QuikChange Lightning Multi Site-Directed Mutagenesis Kit (Agilent Technologies).

### Antibodies

The following antibodies were used for immunoblotting at the indicated dilutions: anti-FLAG-HRP (A8592, Sigma Aldrich, 1:1250), anti-V5 (46–0705, Invitrogen, 1:5000), and anti-GST (27–4577–01, GE Healthcare, 1:2000); anti-p-Tyr-HRP (sc-7020, 1:500), anti-p85 (sc-423, 1:500), anti-Hsp56 (sc-1803, 1:1250), anti-GAPDH (sc-47724, 1:5000), and anti-HA-HRP (sc-7392, 1:500) were purchased from Santa Cruz Biotechnology; anti-ACK (07–757, 1:2000) and anti-ACK p-Tyr284 (09–142, 1:5000) were purchased from Millipore; anti-Histone H3 (ab1791, 1:5000) and anti-pTyr607 p85 (ab182651, 1:1000) were purchased from Abcam. The HRP-conjugated secondary antibodies donkey anti-mouse (sc-2318, 1:2000) and donkey anti-rabbit (sc-2317, 1:5000) were purchased from Santa Cruz Biotechnology. Anti-FLAG (F3165, Sigma Aldrich) was used for immunoprecipitation. If necessary, antibodies were diluted in PBS-0.1% Tween.

### Cell lines

HEK293T cells were cultured in Dulbecco's modified Eagle's medium (DMEM) supplemented with 10% foetal bovine serum and transfected using Lipofectamine 2000. To generate HEK293T cell lines stably expressing HA-wtACK or HA-caACK, Phoenix-AMPHO cells were transfected with pBABE-puro-wtACK or caACK. Retroviral supernatant was collected after 72 h and added directly to HEK293T cells for 16 h with 4 ng/μl polybrene. Infected cells were selected using 1 μg/ml puromycin dihydrochloride. To generate HEK293T cell lines stably expressing FLAG-wt p85β/p85β Y599F, HEK293T cells were transfected with 8 μg pSLIK-hygro-FLAG-wt p85β/p85β Y599F and 4 μg each of the packaging vectors pCMV-VSV-G and pCMV-ΔR8.2 dvpr. After 16 h, viral supernatant was collected and added directly to recipient HEK293T cells for 16 h with 4 ng/μl polybrene. Infected cells were selected using 150 μg/ml Hygromycin B. Transgene expression in each clonal line was confirmed by immunoblotting. Control cell lines were generated in the same way using empty vector but were expanded as a polyclonal cell line.

### Cell proliferation assays

Matched cell lines were chosen that had similar expression levels of the transgene. One to three matched lines of each variant were tested according to availability. Cells were seeded into 6-well plates at a density of 3 × 10^4^ cells per well and incubated at 37 °C, 5% CO_2_. Assays using ACK stable cell lines were performed in DMEM + 0.5% FBS. Assays using p85β cell lines were performed in DMEM + 10% FBS. p85β expression was induced using 1 μg/ml doxycycline (with which all cells were treated), 24 h after seeding. Cells were trypsinized and dispersed using a syringe before being counted using a Countess automated cell counter at specific timepoints. Data were tested for with group significance testing using a two-way ANOVA; ∗*p* <0.05; ∗∗*p* < 0.01.

### Co-immunoprecipitation

Small-scale co-immunoprecipitations were performed using 1 μg antibody cross-linked to Protein G Dynabeads (Life Technologies) with dimethyl pimelimidate. HEK293T cells were grown in 10 cm dishes to 70% confluence and then transfected for 40 h. Cells were then washed in cold PBS and lysed on ice in 0.5-1 ml mammalian cell lysis buffer (50 mM Tris-HCl pH 7.5, 150 mM NaCl, 1 mM EDTA, 1 mM sodium orthovanadate, 1 mM β-glycerophosphate disodium salt hydrate, 1X Mammalian Protease Inhibitor Cocktail (Sigma Aldrich, P8340), 1% Triton X-100). Lysates were centrifuged at 17,000*g* for 20 min at 4 °C, and supernatants were precleared by incubation with 50 μl Dynabeads at 4 °C for 1 h. Once cell lysates had precleared, the lysate was rotated with 50 μl antibody-bound beads for 1 h. The beads were then washed three times with mammalian cell lysis buffer, and protein complexes were eluted using 20 μl 2X LDS sample buffer (Life Technologies), 3M β-mercaptoethanol, mixed with 20 μl PBS.

### Immunoblotting

Proteins were transferred to Immobilon-P PVDF membrane (Millipore) using an X-Cell II Blot Module (Life Technologies) following manufacturer’s instructions. Membranes were blocked in 10% milk dissolved in PBS-0.1% Tween. Protein bands were visualized using enhanced chemiluminescence.

### Cell fractionation

Cells were grown in 10 cm dishes to 70% confluence and then transfected for 40 h. Cells were then washed in PBS, resuspended, and pelleted by centrifugation at 500*g* for 20 min at 4 °C. Cell pellets were then resuspended in 500 μl cold hypotonic buffer (20 mM Tris-HCl pH 7.4, 10 mM NaCl, 3 mM MgCl_2_) and incubated on ice for 15 min. Twenty-five microliter NP-40 alternative was then added and cells were lysed by vortexing for 10 s. Samples were then centrifuged at 500*g* for 20 min at 4 °C to separate the cytoplasmic fraction from intact nuclei and other subcellular structures. The supernatant (cytoplasmic fraction) was removed and stored on ice. The pellet was washed with 500 μl cold hypotonic buffer and then resuspended in 500 μl cell extraction buffer (100 mM Tris-HCl pH 7.4, 100 mM NaCl, 1 mM EDTA, 1 mM EGTA, 0.1% SDS, 1 mM NaF, 2 mM Na_3_VO_4_, 1% Triton X-100, 10% glycerol, 0.5% deoxycholate, 20 mM Na_4_P_2_O_7_, 1 mM PMSF, 1X Mammalian Protease Inhibitor Cocktail). Pellets were incubated on ice for 30 min with vortexing every 10 min. Insoluble debris was cleared from the nuclear-enriched fractions by sonication (2 mm probe, 40% amplitude, 10 s) and centrifugation at 17,000*g* for 30 min at 4 °C. Cytoplasmic and nuclear-enriched fractions were then either analyzed by SDS-PAGE gel directly or used in co-immunoprecipitation studies.

### Expression and purification of recombinant regulatory subunits and domains

Full-length human p85α, p50α, p55α, p85α nSH2 (325–430), p85α cSH2 (614–720), mouse p85β, and p85β cSH2 (603–712) were expressed in *E. coli* as GST fusion proteins. Expression was induced by 1 mM IPTG for 16 h at 20 °C. Cells were harvested and resuspended in MTPBS (150 mM NaCl, 16 mM Na_2_HPO_4_, 4 mM NaH_2_PO_4_ pH 7.3) supplemented with SIGMAFAST Protease Inhibitor Cocktail Tablet (EDTA-free) and 1 mM PMSF, and lysed using an Emulsiflex at 10,000 p.s.i. Lysates were centrifuged at 18,000*g* for 40 min at 4 °C and the supernatant applied to glutathione-agarose beads for 2 h at 4 °C. After washing, GST–full-length p85 fusion proteins were eluted from the beads with 10 mM reduced glutathione. GST–SH2 fusion proteins were subject to HRV 3C cleavage while immobilized on GST beads in MTPBS, 1 mM DTT, 1 mM EDTA. Following cleavage, the SH2 domains were further purified by size exclusion chromatography on an S75 column (GE Healthcare).

Full-length human p55γ and mouse p85β nSH2 (312–425) were expressed as fusion proteins with N-terminal His_6_ and MBP tags. Expression of p55γ fusion protein was induced with 1 mM IPTG for 3 h at 30 °C. Expression of p85β nSH2 fusion protein was induced with 1 mM at 37 °C for 5 h. Cell pellets were resuspended in His-protein lysis buffer (20 mM Tris-HCl pH 7.9, 500 mM NaCl) supplemented with Protease Inhibitor Cocktail (Sigma Aldrich, S8830) and 1 mM PMSF. Cells were lysed and pelleted as described above. A Ni-NTA column was equilibrated with 20 ml Ni-NTA buffer A (20 mM Tris-HCl pH 7.9, 150 mM NaCl, 0.05% sodium azide) and cleared lysate was loaded directly onto the column. The Ni-NTA column was then washed with Ni-NTA buffer A, followed by 2% Ni-NTA Buffer B (20 mM Tris-HCl pH 7.9, 150 mM NaCl, 0.05% sodium azide, 1 M imidazole pH 7.9). Fusion proteins were eluted from the column using a gradient of 2-100% Ni-NTA Buffer B. His_6_-MBP-p85β nSH2 fusion was subject to HRV 3C cleavage and further purification by size exclusion chromatography as described previously.

### Expression and purification of His6-ACK (110–489) and GST-ACK (1–489)

ACK residues 110-489 were cloned into pFastBac-HT and recombinant bacmid was generated using *E. coli* DH10Bac. ACK residues 1-489 were cloned into pDEST15 using Gateway technology. GST-ACK (1–489) was expressed in *E. coli* BL21 Rosetta 2(DE3) cells; His_6_-ACK (110–489) was expressed using the baculovirus/Sf9 cell system. His_6_-ACK (110–489) and GST-ACK (1–489) were purified as previously described (44).

### *In vitro* kinase assays

His_6_-ACK (110–489) or GST-ACK (1–489) was incubated for 10 min at 30 °C in kinase buffer (20 mM Tris-HCl pH 7.5, 10 mM MgCl_2_, 0.5 mM DTT, 0.1 mM sodium orthovanadate) supplemented with 0.5 mM ATP pH 7.5 to promote autophosphorylation. For *in vitro* kinase assays, 0.3 μM autophosphorylated His_6_-ACK (110–489) or GST-ACK (1–489) was added to kinase buffer with 30 μM substrate in a final reaction volume of 50 μl and incubated for 30 min at 30 °C.

### Phosphorylation site mapping using LC-MS/MS

Samples were resolved by SDS-PAGE and visualized using InstantBlue (Expedeon). Bands of interest were excised from the gel, destained, reduced using DTT, and alkylated using iodoacetamide. The protein was then digested with trypsin overnight at 37 °C and then loaded onto an autosampler for automated LC-MS/MS analysis. All LC-MS/MS experiments were performed using a nanoAcquity UPLC (Waters Corp.) system and an LTQ Orbitrap Velos hybrid ion trap mass spectrometer (Thermo Scientific). The peptides were first separated by reverse-phase LC using a Waters reverse-phase nano column (BEH C18, 75 μm i.d. × 250 mm, 1.7 μm particle size) at flow rate of 300 nl/min. The peptides were first loaded onto a precolumn (Waters UPLC Trap Symmetry C18, 180 μm i.d. × 20 mm, 5 μm particle size) in 0.1% formic acid for 5 min at a flow rate of 5 μl/min. The peptides were then eluted from the precolumn and loaded onto the nanoAcquity analytical column. The LC eluant was sprayed into the mass spectrometer using a New Objective nanospray source. The m/z values of eluting ions were measured in the Orbitrap Velos mass analyzer, set at a resolution of 30,000. Data-dependent scans (top 20) were then employed to automatically isolate and generate fragment ions by collision-induced dissociation in the linear ion trap, resulting in the generation of MS/MS spectra. Ions with charge states of 2+ and above were selected for fragmentation. The MS/MS data were processed using Protein Discoverer (version 1.3, ThermoFisher). Briefly, all MS/MS data files were submitted to the Mascot search algorithm (Matrix Science) and searched against the PI3K regulatory subunit 1 sequence, using a fixed modification of carbamidomethyl and variable modifications of oxidation (M) and phospho (Ser, Thr, Tyr). Sites of phosphorylation were then verified manually. The peptide and fragment mass tolerances were set to 25 ppm and 0.8 Da, respectively. A significance threshold value of *p* < 0.05 and a peptide cut-off score of 20 were also applied.

### Fluorescent polarization assays

p85α and CD28 peptides were purchased from Biomatik; p85β peptides were purchased from Cambridge Research Biochemicals. Peptide sequences were as follows: peptides based on p85α, [5-FAM]-TEDQ-[pY]-SLVEDE and [5-FAM]-TEDQYSLVEDE; peptides based on p85β, [5-FAM]-TEDQ-[pY]-SLMEDE and [5-FAM]-TEDQYSLMEDE; CD28 peptide, [5-FAM]-SD-[pY]-MNMTP. Fluorescence polarization experiments were measured on a BMG Labtech Pherastar fluorimeter at 298 K with excitation 485 nm and emission 520 nm. Solutions were made up in black, flat-bottom, 96-well plates (Corning) to a final volume of 30 μl. A peptide titration was used to determine the appropriate concentration of peptide for binding assays. CD28, YSLV, and pYSLV were used at 20 nM. YSLM and pYSLM were used at a final concentration of 40 nM. Anisotropy was calculated from polarization measurements in MARS analysis software and data fitted to a single site binding isotherm in Prism.

### Modeling

Dimer models were generated using the structure of the human p85β monomer from AlphaFold (entry 000,459). Distances were measured between the p85β nSH2 domain and a phosphorylated peptide, using the structure of human nSH2 with a peptide from c-Kit (PDB 2IUH). Residues in the c-Kit peptide sequence that contact the nSH2 were mutated in Pymol to the p85β equivalent (Asn2 to Asp and Met5 to Ala). Close contacts (within 4 Å) were measured for Asp603, pTyr605, Ala606, and Met608 (human p85β numbering) and used as symmetric intermolecular restraints, using the measured distance of the closest two atoms with a 1 Å upper bound. Intermolecular contacts between the SH3 domain and the first Pro-rich region in the other monomer were measured in the same way but using the structure of the Fyn SH3 domain with a p85 peptide (PDB 1A0N). Contacts between the RhoGAP domains were assessed using the structure of the p85α RhoGAP (PDB 1PBW). As the region of the RhoGAP involved in intermolecular contacts is not conserved between p85α and p85β, the contacts were added with a 4 Å restraints and a 1 Å upper bound. The restraints tables were used to calculate dimer models using the HADDOCK2.4 server (45, 46). The linkers between the domains were set to be fully flexible as follow: 83-110 (SH3-RhoGAP), 295-323 (RhoGAP-nSH2), and 598-614 (iSH2-cSH2). The linker between nSH2 and iSH2 was not set to be flexible, since AlphaFold predicts a low error for interactions between these two domains. NCS and symmetry restraints were applied across all residues. A separate HADDOCK run was set up containing the same restraints but with the SH3, PR1, and RhoGAP domain of one monomer missing. NCS and symmetry restraints were added for the regions that were present in both monomers.

## Data availability

All data are contained within the manuscript and supporting information.

## Supporting information

This article contains supporting information.

## Conflict of interest

The authors declare that they have no conflicts of interest with the contents of this article,
