# Supplementary material for: Assembly of nuclear dimers of PI3K regulatory subunits is regulated by the Cdc42-activated tyrosine kinase ACK
Source: J Biol Chem. 2022 Apr 13;298(6):101916. doi: 10.1016/j.jbc.2022.101916 (PMC9127371; doi:10.1016/j.jbc.2022.101916)
Supplement: Supporting information [file mmc1.pdf]

Supporting Information

Assembly of novel, nuclear dimers of the PI3K regulatory subunits is regulated by the Cdc42-activated tyrosine kinase, ACK

Natasha S. Clayton[1†¶], Millie Fox[1†¥], Jose J. Vicenté-Garcia[1‡], Courtney M. Schroeder[1§], Trevor D. Littlewood[1], Jonathon I. Wilde[2∫], Kadalmani Krishnan[1#], Murray J. B. Brown[2], Claire Crafter[3], Helen R. Mott[1*] and Darerca Owen[1]

Figure S1. The interactions between endogenous ACK and p85α in LNCaP95 cells.

Figure S2. Phosphorylation of FLAG-tagged regulatory subunit isoforms by HA-ACK in HEK293T cells.

Figure S3. Identification of ACK phosphorylation sites in GST-p50α.

Figure S4: Proliferation of HEK293T cells expressing ACK and p85β variants.

Figure S5. Localization of pY607F p85α to nuclear enriched fractions.

Supporting Information Figure Legends

Figure S1: The interactions between endogenous ACK and p85α in LNCaP95 cells. LNCaP95 cells were lysed and protein levels assessed using the BCA assay (Pierce™ BCA protein assay kit, Life Technologies) following manufacturer's instructions. Lysates containing equal amounts of proteins were subject to immunoprecipitation with anti-p85α or anti-IgG antibodies. Co-precipitation of endogenous ACK was assessed by western blotting using anti-ACK antibodies. M=Markers. (n=1).

Figure S2: Phosphorylation of FLAG-tagged regulatory subunit isoforms by HA-ACK in HEK293T cells. (A-E) HEK293T cells were harvested 40 hours after transfection with FLAG-tagged regulatory subunit isoforms in the presence and absence of HA-ACK or HA-dACK (kinase-dead). Phosphorylation of FLAG-tagged regulatory subunit isoforms was assessed by western blotting of cell lysates using an antibody raised against the p85 sequence (D-Q-$Y_{(p)}$-S-L) and/or a pan anti-pTyr antibody. Results are representative of at least three independent experiments.

Figure S3: Identification of ACK phosphorylation sites in GST-p50α. (A) Full-length GST-p50α was incubated at 30 °C for 30 minutes with His6-ACK (110-489). Reactions were analysed by SDS-PAGE and phosphorylation of GST-p50α at Y307 was assessed by western blotting using an anti-p85 (pTyr607). The presence of active His6-ACK (110-489) was confirmed by western blotting using an anti-ACK (pY284) antibody. (B) *In vitro* kinase assay reactions for GST-p50α in the presence and absence of His6-ACK (110-489) were resolved by SDS-PAGE and proteins visualized by Coomassie staining. (C) Phosphorylation sites in excised full-length GST-p50α (red box, panel B) were identified by LC-MS/MS. Annotated MS/MS spectrum of the tyrosine phosphorylated peptide KLNEWLGNENTEDQYSLVEDDEDLPHHDEK (m/z 923.65, 4+). Detectable y fragment ions between y13 and y20 are labelled. This can be compared with the non-phosphorylated peptide (D) (m/z 903.65, 4+) in which the m/z values are 40 units lower (corresponding to a mass of 80 Da) from y16, which is indicative of tyrosine phosphorylation.

Figure S4: Proliferation of HEK293T cells expressing ACK and p85β variants. (A) Proliferation of HEK293T cells stably expressing wtACK and caACK in 1% FBS. HEK293T cells stably expressing empty vector (control) or HA-tagged wtACK or caACK were seeded at a density of 3 x 10^4 cells per well. Cells were plated in replicates of 3 and live cell counts were obtained at the times indicated. Data is plotted as fold change over t=0 (seeding density). Data points from three independent experiments are shown. Data are represented on the right in a bar chart with floating bars showing minimum and maximum values, and with group significance testing performed by a two-way ANOVA; *, $p < 0.05$; **, $p < 0.01$. (B) Proliferation of HEK293T cells stably expressing empty vector (control), wt p85β or Y599F p85β. HEK293T cells stably expressing inducible FLAG-tagged wt p85β or p85β Y599F were seeded at a density of 3 x 10^4 cells per well and incubated at 37 °C for 16 hours. All cells were then treated with 1μg/mL doxycycline and incubated at 37 °C for the times indicated before live cell counts were taken. Data are plotted as fold change over the cell count at 24 hours post doxycycline treatment. Certain cell lines were treated with the ACK inhibitor AIM-100 (8 μM) as indicated. Data points from three independent experiments are shown. Error bars represent SD. Data are represented on the right in a bar chart with floating bars showing minimum and maximum values, and with group significance testing performed by a two-way ANOVA; *, $p < 0.05$; **, $p < 0.01$.

Figure S5: Localization of pY607F p85α to nuclear enriched fractions. wt p85α and Y607F p85α were expressed alone or co-expressed with ACK in HEK293T cells. Cells were lysed and separated into cytoplasmic and nuclear-enriched fractions. Fractions were analysed by western blotting using the antibodies indicated (left hand panels). Fractionation was judged using anti-Hsp56 to indicate cytoplasmic extracts and anti-Histone H3 to indicate nuclear-enriched fractions. Samples of whole cell lysates (WCL) were quantified for protein levels and equal amounts of lysate protein was analysed by western blotting, to show total levels of proteins using the antibodies indicated and are shown in the right hand panels. Results are representative of at least three independent experiments.
