# Supplementary figures and images for: Assembly of nuclear dimers of PI3K regulatory subunits is regulated by the Cdc42-activated tyrosine kinase ACK
Source: J Biol Chem. 2022 Apr 13;298(6):101916. doi: 10.1016/j.jbc.2022.101916 (PMC9127371; doi:10.1016/j.jbc.2022.101916)

## Supplementary Figure 1

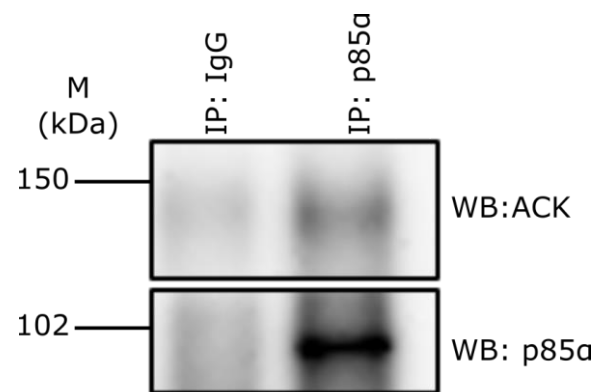

Supplement: Supplemental Figure S1 [file mmc2.pdf]

Supplementary Figure 2

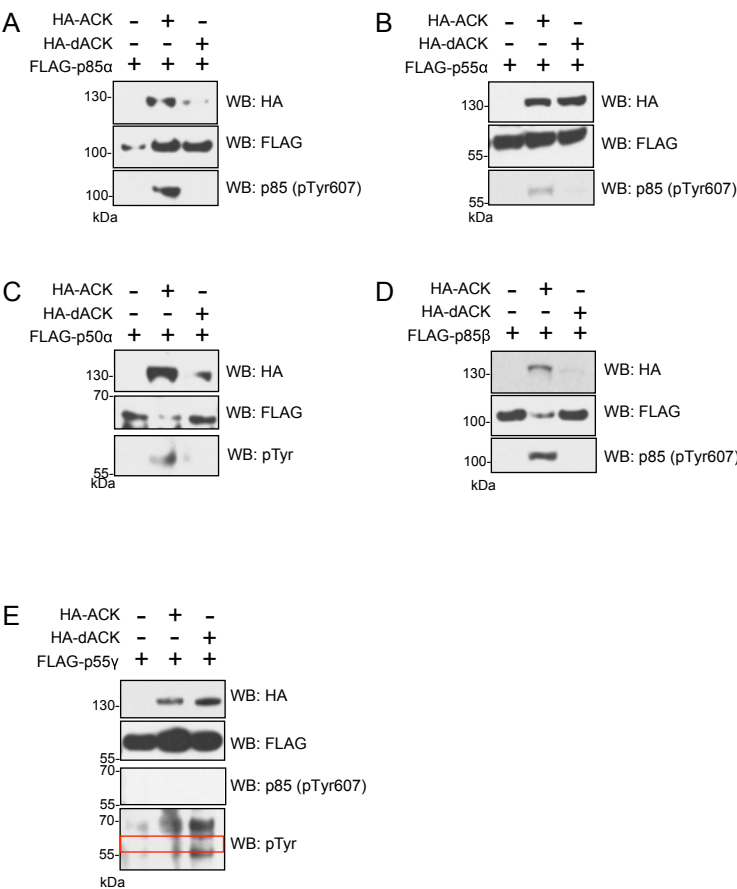

Supplement: Supplemental Figure S2 [file mmc3.pdf]

Figure S3

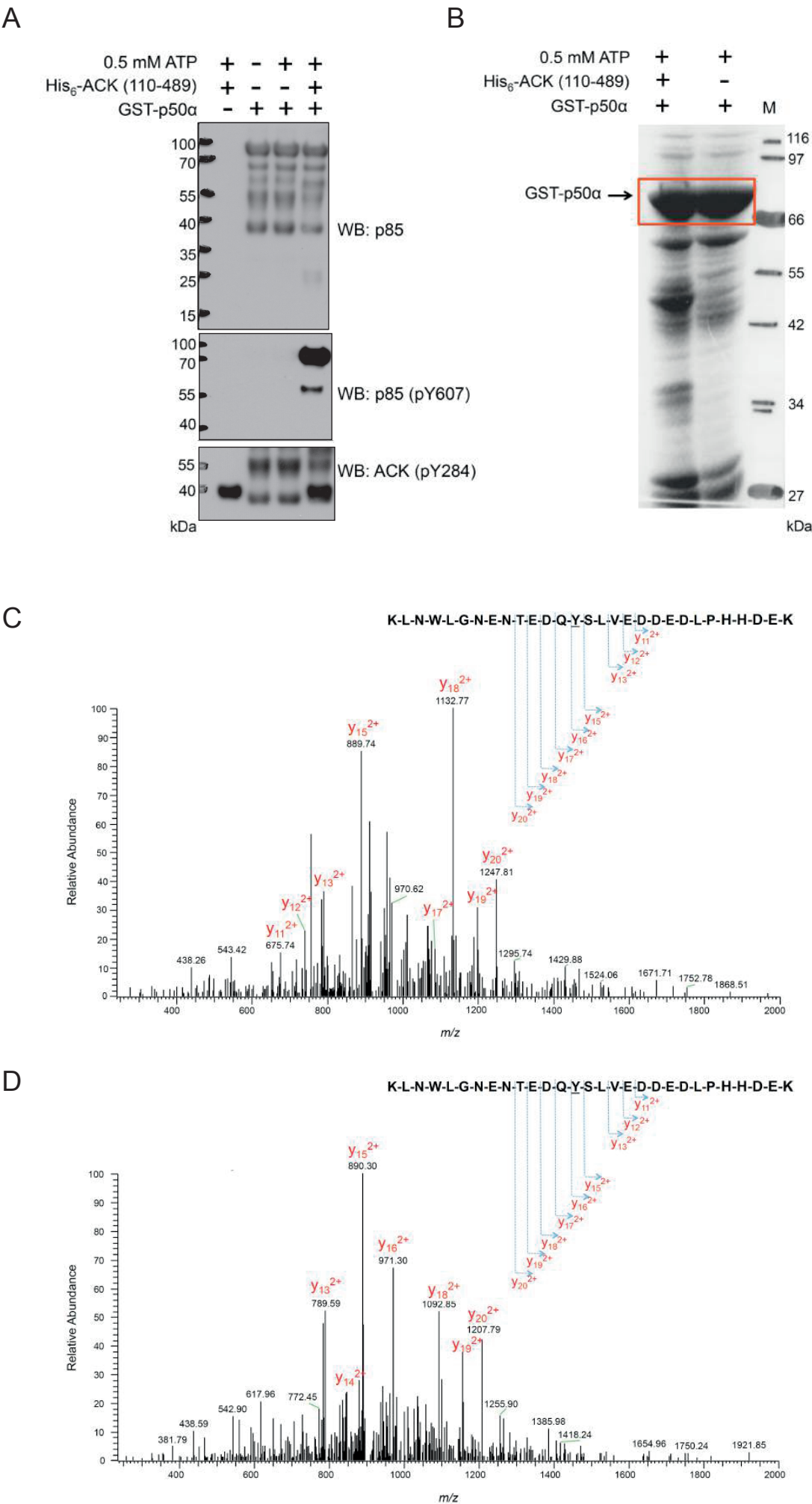

Supplement: Supplemental Figure S3 [file mmc4.pdf]

Figure S4

A

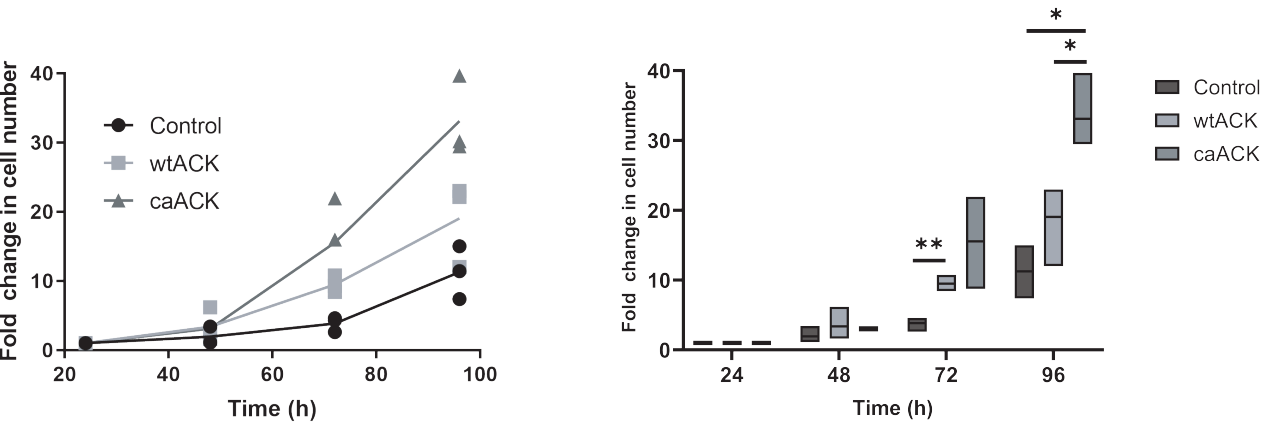

B

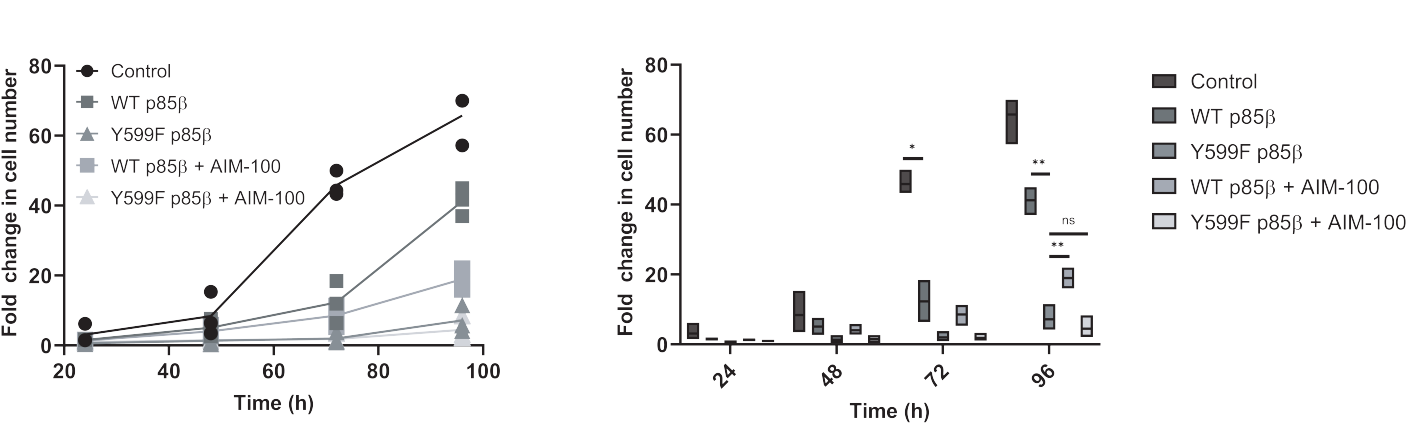

Supplement: Supplemental Figure S4 [file mmc5.pdf]

Figure S5

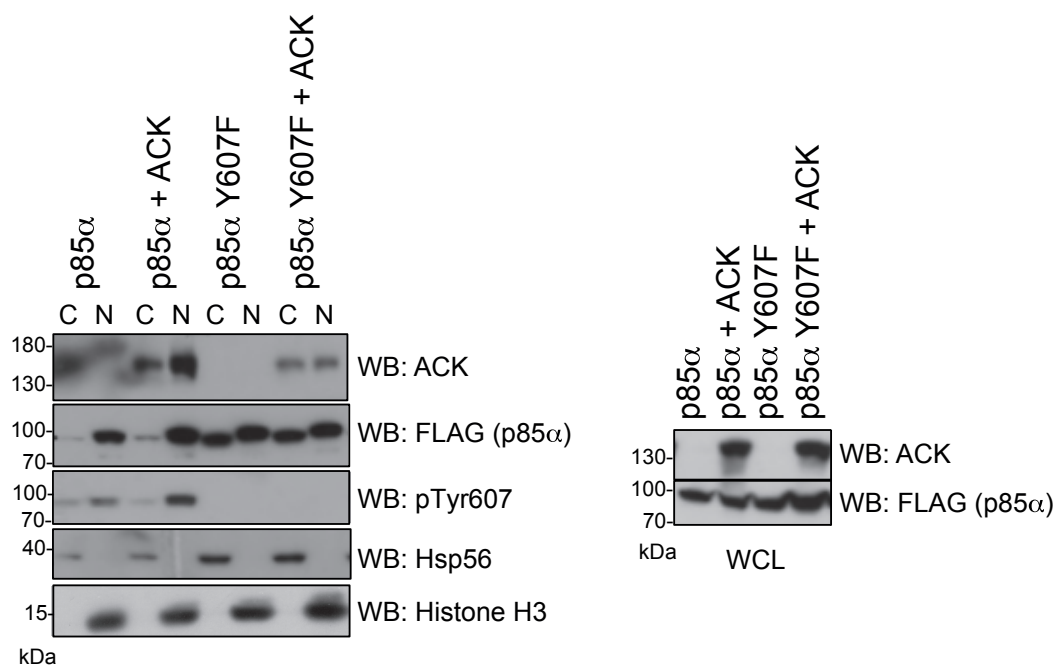

Supplement: Supplemental Figure S5 [file mmc6.pdf]
